# A novel FHOD3 splice-site variant in a Chinese family with hypertrophic cardiomyopathy: a case report

**DOI:** 10.3389/fcvm.2026.1809502

**Published:** 2026-05-29

**Authors:** Bing-Yang Zhou, Ying-Yi Zhang, Ning Ren, Jie Geng

**Affiliations:** 1 Department of Cardiology, Chest Hospital, Tianjin University, Tianjin, China; 2 Department of Cardiology, Tianjin Chest Hospital, Tianjin, China; 3 Department of Cardiology, Tianjin Key Laboratory of Cardiovascular Emergency and Critical Care, Tianjin Municipal Science and Technology Bureau, Tianjin, China

**Keywords:** case report, FHOD3, gene mutation, hypertrophic cardiomyopathy, non-sarcomeric proteins

## Abstract

Hypertrophic cardiomyopathy (HCM) predominantly manifests as an autosomal dominant disorder, with approximately 60% of cases carrying pathogenic or likely pathogenic genetic variants. Although more than 90% of pathogenic variants in HCM patients occur in eight core sarcomeric protein-encoding genes, some cases may be linked to variants in additional genes. Formin Homology 2 Domain Containing 3 (FHOD3), which encodes a non-sarcomeric protein, has been associated with the pathogenesis of HCM. Here, we report a young male patient with asymmetric myocardial hypertrophy assessed by echocardiography, who was asymptomatic. Whole-exome sequencing was performed on the proband, and candidate variants were validated by Sanger sequencing. A heterozygous putative splice-site variant (c.1286 + 2delT) in FHOD3 gene was identified in the proband, as well as in his mother and brother. This variant has mainly been reported in Chinese cohorts and may represent a population-enriched variant, although further studies are required to confirm this observation.

## Introduction

The prevalence of hypertrophic cardiomyopathy (HCM) was estimated at 1:500 in previous years, but recent advances in cardiac magnetic resonance (CMR) imaging and genetic analysis suggest that prevalence may be as high as 1:200 (1). The primary pathogenesis of HCM involves variants in genes encoding sarcomeric proteins or proteins associated with sarcomere structure. HCM predominantly manifests as an autosomal dominant disorder, with approximately 60% of cases carrying pathogenic or likely pathogenic genetic variants (2). However, the etiology and pathogenesis of HCM remain incompletely understood in some cases, highlighting the need to identify novel pathogenic mutations. Although more than 90% of pathogenic variants in HCM patients occur in eight core sarcomeric protein-encoding genes, a small number of cases may be linked to variants in additional genes. In 2013, Formin Homology 2 Domain Containing 3 (FHOD3), which encodes a non-sarcomeric protein, was found to be associated with the pathogenesis of HCM (3). In 2018, Ochoa et al. reported that FHOD3 mutations co-segregated with HCM across 17 families, with a combined logarithm of the odds score of 7.92, indicating very strong segregation (4). The authors concluded that FHOD3 represents a novel pathogenic gene in HCM, implicated in approximately 1%–2% of all cases. However, few studies reported the pathogenicity of FHOD3 variants in HCM patients.

Here, we describe a FHOD3 gene variant in a Chinese family, in which the proband was clinically diagnosed with HCM but remained asymptomatic.

## Case presentation

The proband was an 18-year-old man diagnosed with myocardial hypertrophy during a routine physical examination. However, he had not experienced chest pain, shortness of breath, palpitations, dizziness, or syncope. He denied a history of hypertension or diabetes mellitus. He was a student with no history of smoking or drinking. He had no family history of cardiac diseases or sudden death at a young age. His height was 1.78 m, weight 78 kg, and body mass index 24.62 kg/m^2^. His systolic blood pressure was 119 mmHg and diastolic blood pressure was 69 mmHg. His levels of B-type natriuretic peptide and high-sensitive troponin T were normal. Aldosterone, renin, and their ratio were all within the normal ranges. Immunofixation electrophoresis revealed no abnormal bands, and quantification of serum free light chains was normal.

**Figure 1:**
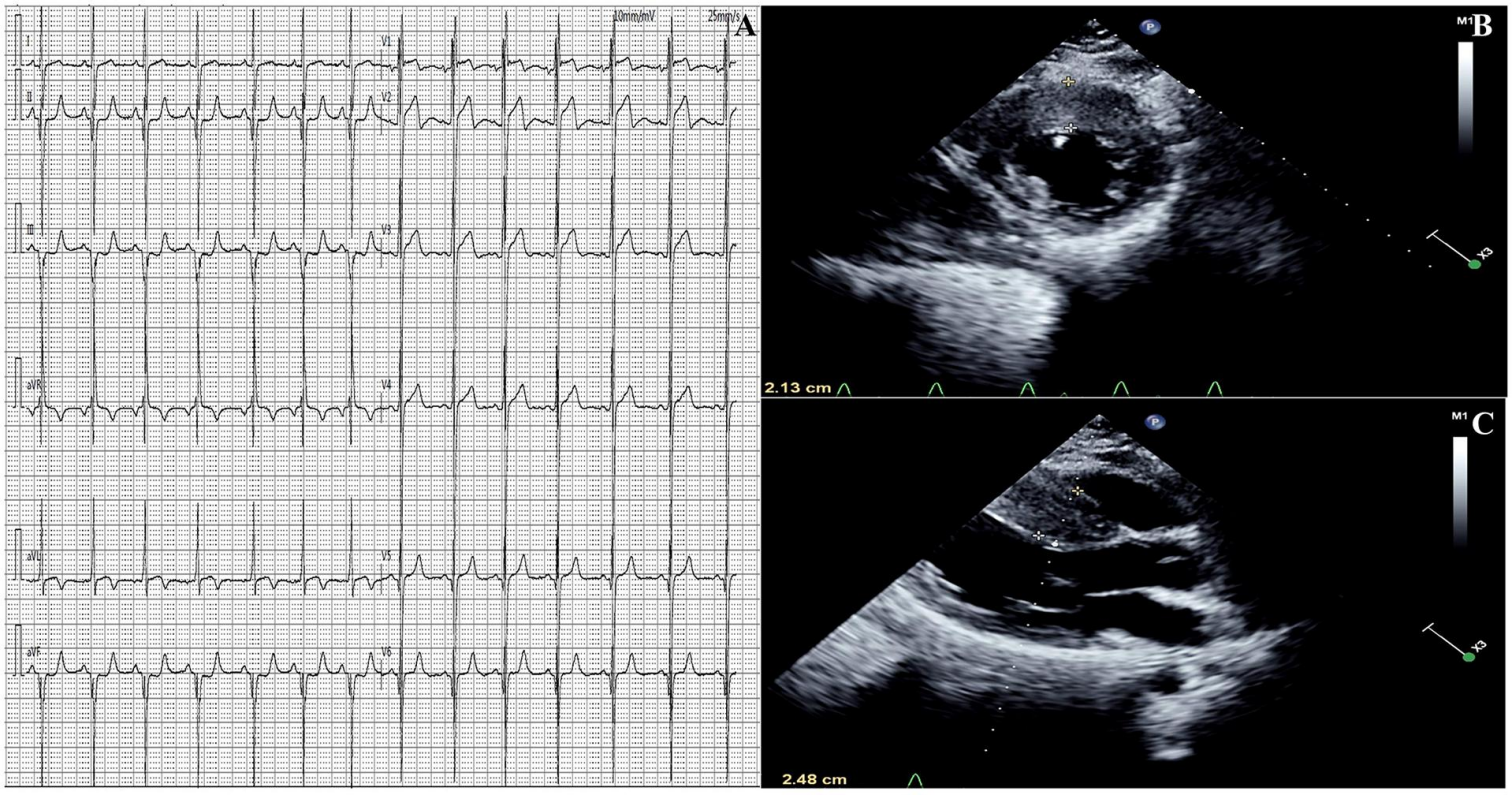
**(A)** The 12-lead ECG of the proband: sinus rhythm with complete right bundle branch block, left ventricular high voltage, and left axis deviation. **(B,C)** Echocardiography showed the anterior interventricular septal thickness of the proband ranging from 2.13 to 2.48 cm.

**Figure 2:**
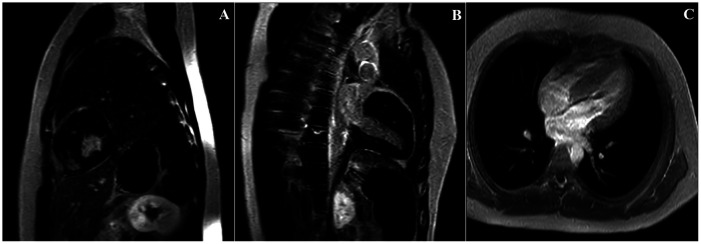
Late gadolinium enhancement image of the proband: **(A)** short-axis image, **(B)** two-chamber view, and **(C)** four-chamber view.

The 12-lead electrocardiogram (ECG) demonstrated sinus rhythm with complete right bundle branch block, left ventricular high voltage, and left axis deviation (Figure 1A). Echocardiography performed by an experienced chief physician revealed a left atrial inner diameter of 3.1 cm, left ventricular end-diastolic dimension of 4.0 cm, right atrial diameter of 3.4 cm, and left–right diameter at the base of the right ventricle of 3.0 cm, indicating normal inner atrial and ventricular diameters. Echocardiography showed a left ventricular ejection fraction (LVEF) of 66% and normal pulmonary artery systolic pressure. Asymmetric myocardial hypertrophy was observed: The left ventricular posterior wall thickness was 0.87 cm, while the anterior interventricular septal thickness ranged from 2.13 to 2.48 cm (Figures 1B,C). On stress echocardiography, the left ventricular outflow tract demonstrated a flow velocity of 1.55 m/s, generating a pressure gradient of 10 mmHg. Ultrasound speckle-tracking imaging revealed decreased longitudinal strain of the left ventricular anterior wall, measured at −17.8%. Further examinations were performed to identify the cause of myocardial hypertrophy. CMR imaging confirmed myocardial hypertrophy most prominently in the anterior and interventricular septal walls, consistent with echocardiographic findings. Late gadolinium enhancement in the anterior wall of the left ventricle indicated myocardial fibrosis (Figures 2A–C). Fabry disease was excluded based on normal *α*-galactosidase A enzymatic activity. HCM is defined as unexplained left ventricular wall thickness ≥1.5 cm in adults. Therefore, the young man was clinically diagnosed with HCM.

**Figure 3:**
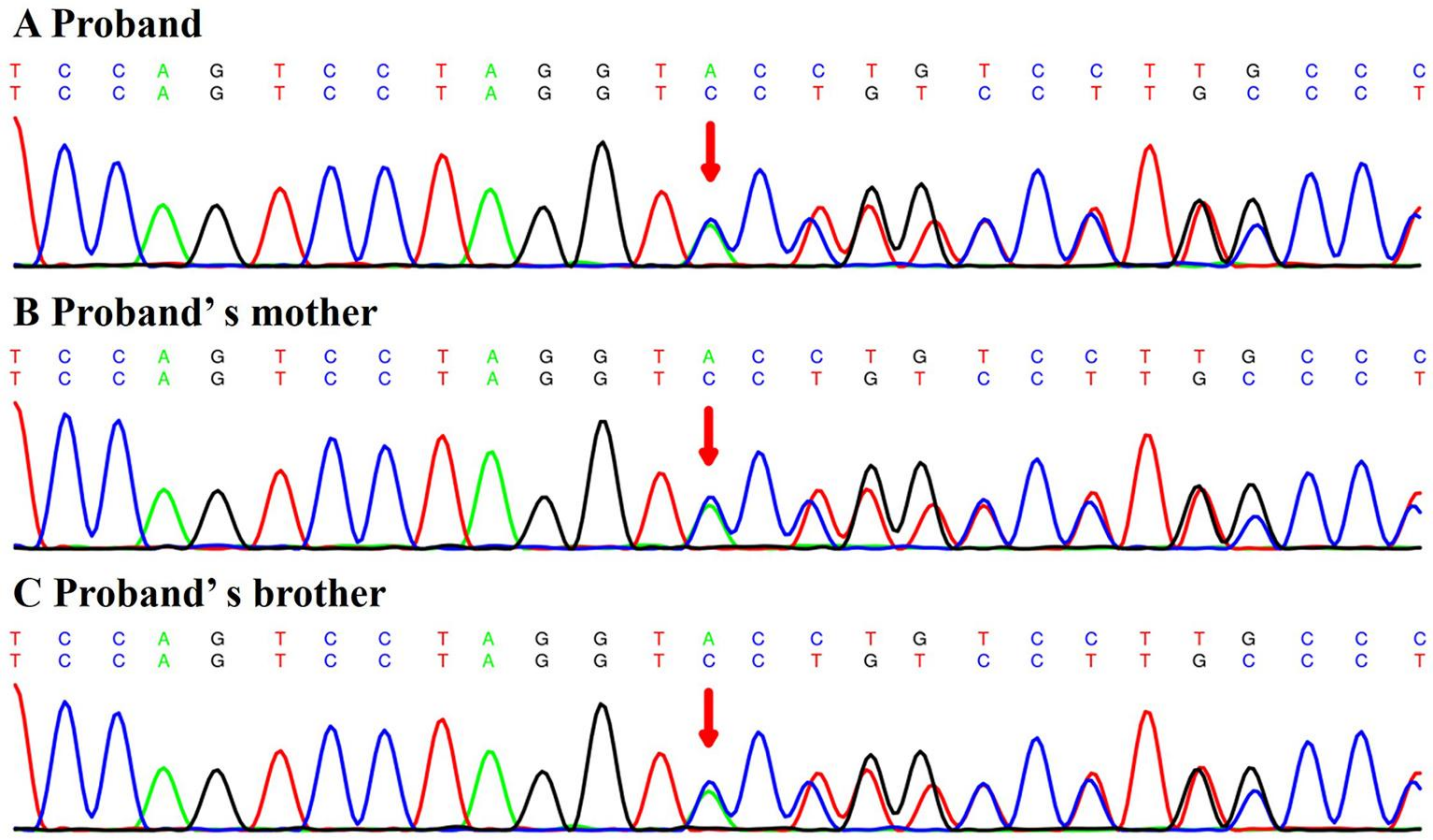
Sanger sequencing showed a heterozygous putative splice-site variant (c.1286 + 2delT) in FHOD3: **(A)** the proband, **(B)** the proband's mother, and **(C)** the proband's brother.

Following ethical approval and informed consent, whole-exome sequencing (WES) was performed on the proband, and candidate variants were validated by Sanger sequencing. Whole-exome sequencing was performed on an Illumina NovaSeq 6000 System using paired-end 2 × 150 bp reads. The sequencing achieved coverage of 99.08% for the target regions, with an average depth of 91.84 ×, ensuring sufficient depth for reliable variant detection. To reduce potential technical artifacts, heuristic quality-control filters were applied during variant calling. Variants were excluded if they met any of the following criteria: missingness >10%, minimum read depth ≤15 reads, allele balance ≤20%, genotype quality <30, or mappability <1 based on 150-bp fragments. Variants were filtered using standard bioinformatic pipelines, including 1000 Genomes, ExAC, ESP6500, ClinVar, HGMD, and OMIM databases. The sequencing panel for HCM included all genes associated with HCM, such as MYBPC3, MYH6, TNNT2, TNNT3, and FHOD3. A heterozygous putative splice-site variant (c.1286 + 2delT) in the FHOD3 gene was identified in the patient, who was clinically diagnosed with HCM (Figure 3A). Based on the currently available evidence and American College of Medical Genetics and Genomics/Association for Molecular Pathology criteria, the FHOD3 c.1286 + 2delT variant is best classified as likely pathogenic. SpliceAl software was used for splicing prediction (score: 0.96, >0.2 affects splicing), indicating the potential impact of the c.1286 + 2delT variant on splicing.

**Figure 4:**
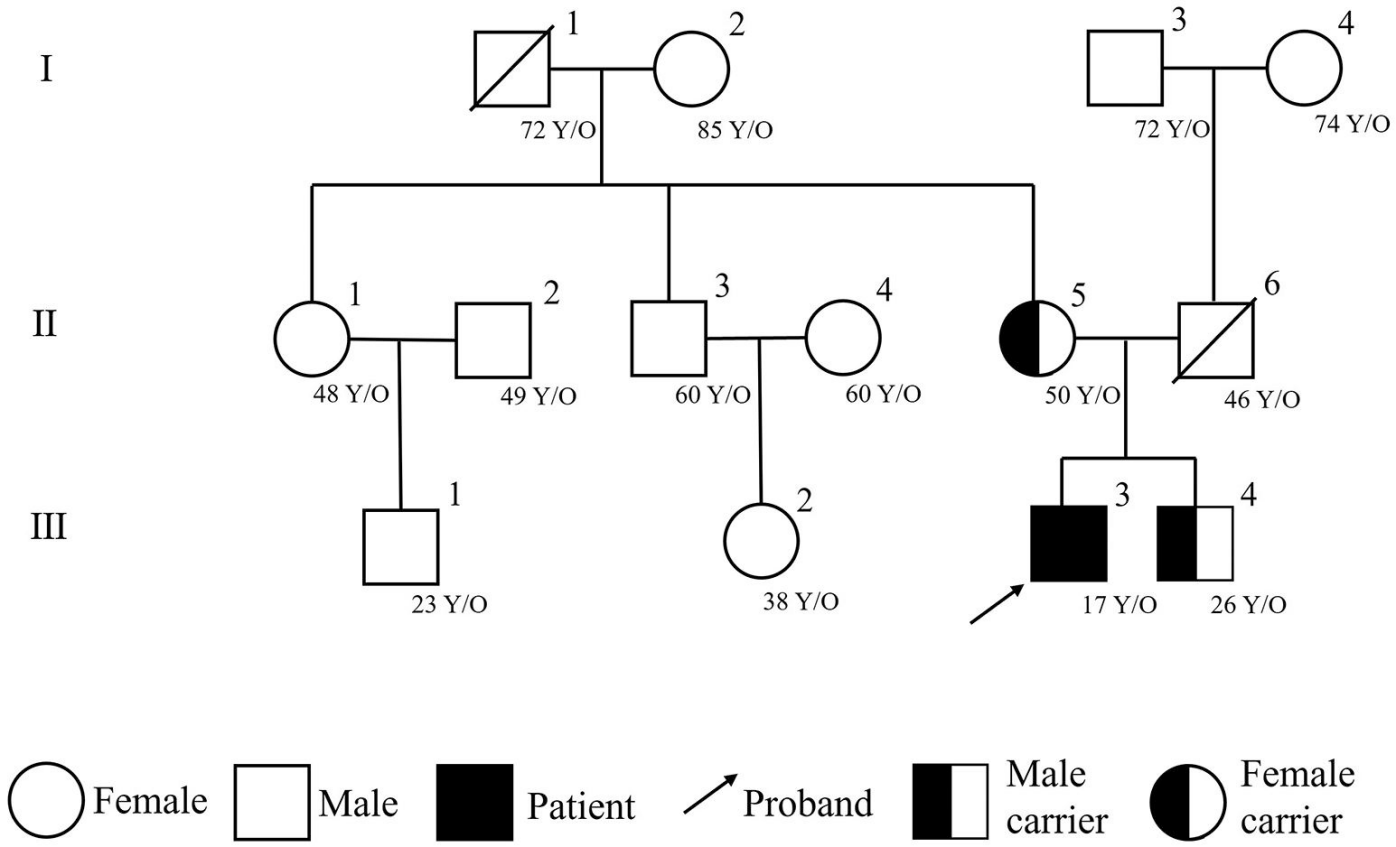
The family pedigree chart of the patient.

The family pedigree of the patient is shown in Figure 4. His mother (II-5) and elder brother (III-4) also underwent genetic analysis and echocardiography. Although both immediate family members (II-5 and III-4) carried the FHOD3 c.1286 + 2delT variant (Figures 3B,C), they did not exhibit increased ventricular wall thickness. The proband's father (II-6) was deceased and had no history of cardiac disease. The proband's maternal aunt (II-1), maternal uncle (II-3), and maternal grandmother (I-2) had normal ventricular wall thickness, although they did not undergo genetic testing. The proband's maternal grandfather (I-1) died of chronic obstructive pulmonary disease at the age of 72 years.

## Discussion

Mutation in genes encoding sarcomeric proteins—including MYH7, MYBPC3, TNNT2, TNNT3, MYL2, MYL3, TPM1, and ACTC1—have been identified as pathogenic for HCM (5). However, some HCM patients do not carry variants of these genes. FHOD3 gene mutations are rare and were first found to be associated with HCM in 2018 (4). Subsequently, the FHOD3 gene has been recommended for routine testing in HCM patients. Pathogenic FHOD3 mutations are predominantly non-truncating in nature, including missense, splice-site, and in-frame indels, and are concentrated within two distinct structural domains: exon 12 and the coiled-coil domain encoded by exons 15 and 16 (4, 6, 7).

Consistent with previous studies, through WES, we identified a c.1286 + 2delT variant in the FHOD3 gene in a Chinese HCM patient and his first-degree relatives. The FHOD3 mutation in our case was in exon 11. The proband was clinically diagnosed with HCM but remained entirely asymptomatic. The variant was absent from population frequency databases (1000 Genomes: none, ESP6500: none, ExAC: none, gnomAD: none). The predicted result using SpliceAl software was 0.96 (range 0–1, >0.2 affects splicing), indicating that the variant may affect splicing. This variant has not been reported in the ClinVar database, while the HGMD database classifies it as a disease-causing mutation.

Formins, encoded by genes located at chromosomal position 18q12.2 in humans, are a family of cytoskeletal regulators that affect both actin and microtubule dynamics. This family includes the FHOD subfamily, which comprises two human members: FHOD1 and FHOD3. The role of FHOD3 in sarcomerogenesis was observed by replating-induced pluripotent stem cell-derived cardiomyocytes (8). FHOD3 is essential for maintaining cardiac contractile structures through its critical role in sarcomere assembly and maintenance (9). FHOD3 has three major protein-coding transcripts in RefSeq/Ensembl: NM_025135.5 (isoform 1), NM_001281739.3 (isoform 2), and NM_001281740.3 (isoform 3). All three transcripts encode protein isoforms that contain the FH2 domain, which is critical for actin polymerization and sarcomere organization in cardiomyocytes. The c.1286 + 2delT variant lies in the donor splice site of intron 11 of this transcript, within the region encoding the FH2 domain. SpliceAI predicts a high probability (0.96) of disrupted splicing, likely leading to exon skipping, intron retention, or cryptic splice activation. These disruptions are expected to impair the function of isoform 3 and support the pathogenicity of the variant. The FHOD3 variant was also found to be related to dilated cardiomyopathy (DCM). A Japanese study analyzed 48 familial DCM patients and found a missense variant of Tyr1249Asn in FHOD3 in an adult-onset patient (10).

FHOD3 gene variants have been recognized to be associated with a relatively mild clinical phenotype in HCM patients. In a study of patients with HCM in the Balkan region, FHOD3 variants were found to be the second most common genetic cause, with a higher prevalence than reported in other populations (11). The authors conducted exome sequencing in 134 probands with HCM and discovered the variant c.1646 + 2T > C in FHOD3 in eight probands. They also reported that half of the individuals were asymptomatic and without adverse cardiac events. Consistent with previous data, Chinese HCM patients with FHOD3 gene mutation were also reported to have mild clinical symptoms, such as shortness of breath after running (12). In our case, although the proband had extremely abnormal interventricular septal thickness of 4.57 cm, he presented with transient palpitation. Genetic testing revealed an FHOD3 variant, but the proband was asymptomatic, and his mother and brother showed no evidence of increased interventricular septal thickness on imaging and or any symptoms typical of HCM. This observation suggests incomplete penetrance within the family. Such findings are not unexpected in HCM, which is characterized by age-dependent penetrance and variable expressivity. Genotype-positive individuals may remain phenotype-negative at the time of evaluation, or may develop only mild, delayed, or subclinical manifestations during follow-up. Therefore, the absence of ventricular hypertrophy in the proband's mother and brother does not exclude a contributory role of the FHOD3 c.1286 + 2delT variant, but indicates that its phenotypic effect may not be fully penetrant in all carriers. In addition, variable clinical expression among carriers of the same variant has been widely recognized in HCM. It is also possible that this variant acts as a modifier of disease susceptibility or severity, rather than as a fully penetrant monogenic cause in every individual. The current understanding of variable penetrance in FHOD3-associated HCM remains incomplete, and further studies are necessary to explore the underlying mechanisms that contribute to this clinical heterogeneity.

Notably, the FHOD3 c.1286 + 2delT variant has been reported in a previous Chinese study which analyzed FHOD3 in 1,000 HCM patients and found 25 missense variants and 2 truncating variants (7). The truncating variant (c.1286 + 2delT)) was identified in four HCM patients. This variant has mainly been reported in Chinese cohorts and may represent a population-enriched variant, although further studies are required to confirm this observation.

## Conclusion

The identification of pathogenic or potentially pathogenic variants is important for genetic counseling, prognostic assessment, and clinical management of at-risk relatives of patients with HCM. In this study, we reported a heterozygous putative splice-site variant, c.1286 + 2delT, in FHOD3 in a Chinese family with HCM. Our findings support the possible involvement of this variant in the HCM phenotype, while also highlighting the likelihood of incomplete penetrance and variable expressivity among carriers. FHOD3 variants should be considered during comprehensive genetic testing in patients with suspected HCM. Further functional studies and longitudinal family follow-up are needed to clarify the pathogenic role of this variant.

## Data Availability

The original contributions presented in the study are included in the article, further inquiries can be directed to the corresponding author upon reasonable request.
